# TADA: taxonomy-aware dataset aggregator

**DOI:** 10.1093/bioinformatics/btad742

**Published:** 2023-12-07

**Authors:** Emil Hägglund, Siv G E Andersson, Lionel Guy

**Affiliations:** Molecular Evolution, Department of Cell and Molecular Biology, Science for Life Laboratory, Biomedical Centre, Uppsala University, SE-751 24 Uppsala, Sweden; Molecular Evolution, Department of Cell and Molecular Biology, Science for Life Laboratory, Biomedical Centre, Uppsala University, SE-751 24 Uppsala, Sweden; Department of Medical Biochemistry and Microbiology, Science for Life Laboratory, Biomedical Centre, Uppsala University, SE-751 23 Uppsala, Sweden

## Abstract

**Summary:**

The profusion of sequenced genomes across the bacterial and archeal domains offers unprecedented possibilities for phylogenetic and comparative genomic analyses. In general, phylogenetic reconstruction is improved by the use of more data. However, including all available data is (i) not computationally tractable, and (ii) prone to biases, as the abundance of genomes is very unequally distributed over the biological diversity. Thus, in most cases, subsampling taxa to build a phylogeny is necessary. Currently, though, there is no available software to perform that handily. Here we present TADA, a taxonomic-aware dataset selection workflow that allows sampling across user-defined portions of the prokaryotic diversity with variable granularity, while setting constraints on genome quality and balance between branches.

**Availability and implementation:**

TADA is implemented as a snakemake workflow and is freely available at https://github.com/emilhaegglund/TADA.

## 1 Introduction

Taxon sampling is the selection of a representative set of taxa to be used in evolutionary analysis to understand the evolution of the entire clade from which the taxa have been sampled. In general, more data is beneficial to phylogenomic and comparative genomic analysis, as including a greater fraction of the diversity increases the resolution of the analyses. However, affordable genome sequencing caused an overrepresentation of clinically and agriculturally relevant taxa (Wu *et al.* 2009). Similarly, metagenomics databases are skewed toward abundant taxa, and have systematic biases (McLaren *et al.* 2019).

The number of possible tree topologies increases with the number of taxa in a super-exponential way (Felsenstein 1978), making it key to limit the size of the dataset to save computation time when inferring phylogenies. It thus is important to reduce redundancy in a dataset but ensure that diversity is well covered. Also, due to compositional biases and differences in the evolutionary rates among taxa, the way taxa are sampled for a dataset might affect the resulting phylogeny. An automated process to generate datasets for this type of analysis can help test the robustness of evolutionary hypotheses using datasets with different taxa compositions.

Several tools and workflows, such as GToTree (Lee 2019), anvi’o (Eren *et al.* 2021), and GEN-ERA toolbox (Cornet *et al.* 2022) have been designed to automate the process of generating phylogenomic trees from provided datasets. Sub-sampling large datasets may be performed by clustering genomes with e.g. FastANI (Jain *et al.* 2018), which uses average nucleotide identity (ANI) to compare genomes. However, the process of selecting one representative per cluster, and processing the data downstream, must be done manually.

The Perl-based phyloSkeleton (Guy 2017) is, to the best of our knowledge, the only tool that can build these datasets, and provides the user with a concatenated alignment of marker proteins for the included taxa. However, phyloSkeleton is slow, cannot sample arbitrary numbers of genomes per clade, and does not offer the ability to parse data from GTDB (Parks *et al.* 2022). PATS (Powell and Battistuzzi 2022) can be used to test the effect of taxon sampling by iteratively removing taxa or groups of taxa and calculating new phylogenies but also requires an initial provided dataset.

Here, we present a snakemake workflow (Mölder *et al.* 2021), to assemble bacterial and archaeal genome datasets for comparative genomics and phylogenetic analysis purposes, based on taxonomy- and phylogenomic-aware sampling. Based on user-defined configuration and sampling schemes, the workflow constructs and downloads datasets ready for downstream analysis.

## 2 Approach and features

**Figure 1.**
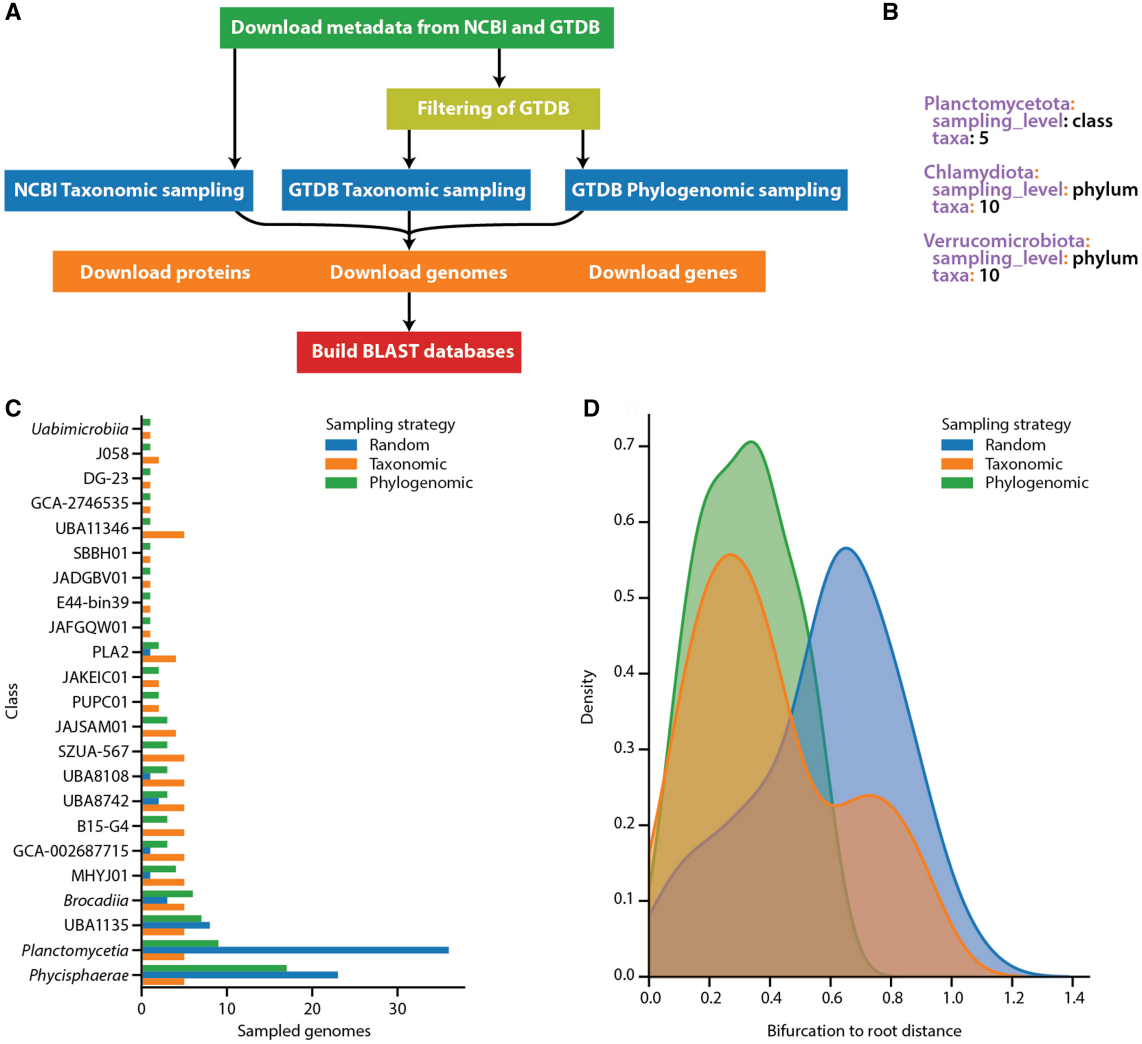
(A) Overview of the TADA workflow. (B) An example of a sampling scheme used for taxonomic sampling with a denser sampling of Planctomycetota compared to the rest of the PVC-superphylum. (C) Number of sampled genomes per class from the *Planctomycetota* phylum in datasets created by random sampling (blue), TADA taxonomic sampling (orange), and TADA phylogenomic sampling (green). A more even distribution across classes is likely to better cover the diversity in the sampled taxon. (D) Distribution of the distances between internal nodes (bifurcations) and the root for maximum-likelihood phylogenies of the *Planctomycetota* phylum based on random sampling (blue), TADA taxonomic sampling (orange), and TADA phylogenomic sampling (green). Tip-to-root distances (not shown) were very similar for all three distributions. Shorter and more evenly distributed distances from internal nodes to the root reduce the risk for artifacts, among others long-branch attraction, when inferring phylogenies.

TADA generates datasets for evolutionary and comparative genomic studies of bacterial and archaeal genomes. Given a few user-defined options and rules, it downloads taxonomic and phylogenomic information from publicly available sources and then performs a sampling procedure. After sampling, TADA can also download genomic data and construct BLAST databases for the sampled genomes. The sampling step can either be based on taxonomic information or phylogenomic information (Fig. 1A).

Taxonomic sampling can be configured to use the taxonomy provided by GTDB or by NCBI Taxonomy. In this approach, TADA relies on a user-created file containing a sampling scheme with one or several sampling criteria, each defined by the name of the taxon to sample from (e.g. “*Enterobacterales*”), the taxonomic level to sample at (e.g. “genus”), and the number of genomes to sample from each clade at the given taxonomic level. A sampling scheme can contain several sampling criteria to sample different numbers of genomes for different parts of the taxonomy. In those cases, TADA will perform a hierarchical sampling by first sampling taxa from the lowest taxonomic rank and continuing to higher ranks, excluding clades it already has sampled from (Fig. 1B).

In the cases where there are intermediate taxonomic levels between the taxon to sample from (e.g. the class *Enterobacterales*) and the taxonomic level to sample at (e.g. sample from each genus), sampling probabilities are adjusted so that each intermediate taxon (e.g. the families *Enterobacteraceae*, *Yersiniaceae*) has an equal probability of being sampled. Furthermore, the user can also provide a file containing assembly accessions for required genomes which should be included in the dataset.

Phylogenomic sampling aims at even better retaining taxon diversity in the sample by pruning GTDB’s bacterial and archaeal phylogenies down to a user-set number of genomes. Optionally, the user can specify that only a part of the tree should be sampled. The pruning is done iteratively by finding the leaf pair with the shortest evolutionary distance and removing one of the taxa from this pair. The default setting is to keep the taxon with the shortest branch to remove fast-evolving organisms, as these are often more prone to introduce artifacts in phylogenies. The workflow can also be configured to randomly select a taxon from the leaf pair or keep the taxon with the longest branch.

When GTDB is used as the source, for taxonomic or phylogenomic sampling, additional criteria such as completeness and contamination estimates can be used to exclude low-quality assemblies. When NCBI taxonomy is used, the user can select either GenBank or RefSeq as the source database. After sampling, irrespective of the method used, TADA can also download genomes, genes, and proteomes for the sampled taxa, using NCBI Datasets. The workflow will run Prokka (Seemann 2014) to annotate genes and proteins whenever an annotation is unavailable through NCBI. Finally, the user can also select to create local alignment databases, for use with NCBI BLAST (Altschul *et al.* 1990) or Diamond (Buchfink *et al.* 2014). The downloaded genomes and proteomes can be then used to generate orthologous gene clusters, e.g. with OrthoFinder (Emms and Kelly 2019). The clusters may then in turn be used for phylogenomic reconstructions. Further examples of user cases can be found in TADA’s documentation, available at the GitHub repository page.

## 3 Implementation

TADA is implemented as a snakemake workflow with additional scripts written in Python. The workflow is accompanied by an environmental file to create a Conda environment for the execution of the workflow and additional software used by the workflow is also handled by Conda.

The pruning in the phylogenomic sampling step is performed with the ETE3-package (Huerta-Cepas *et al.* 2016). Other software like Treemmer (Menardo *et al.* 2018) can be used to prune phylogenies. However, Treemmer is too computationally intensive to handle the very large phylogenies provided by GTDB. This is due to Treemmer identifying leaf pairs and calculating the distance between them after each pruning step. TADA reduces this runtime by only updating the distance for the newly created leaf-pair after each pruning step. TADA was up to 10-fold faster than Treemmer to prune GTDB’s archaeal tree (Supplementary Fig. S1).

## 4 Usage

Below, we highlight the usage of the two different sampling approaches in TADA to create datasets for a phylum with a skewed abundance of species.

### 4.1 Taxonomic and phylogenomic sampling of the *Planctomycetota* phylum

In GTDB r214, the *Planctomycetota* phylum includes 2450 taxa divided into 28 classes. Most of them (78%) are located in the two classes of *Planctomycetia* and *Phycisphaera*. Thus, a phylogeny of the *Planctomycetota* using all species or a random subsampling will be highly unbalanced. Using TADA we constructed three datasets of high-quality genomes and metagenome-assembled genomes of the *Planctomycetota* phylum, one using taxonomic sampling, a second using phylogenomic sampling, and a third dataset using random sampling. A rough phylogeny based on a concatenated alignment of the same 120 marker genes used by GTDB was constructed for each dataset. See Supplementary Information for detailed methods.

The dataset constructed by random sampling only contains nine of the classes from the phylum and has a large overrepresentation of species from *Planctomycetia* and *Phycisphaera* class (Fig. 1C), leading to fewer and longer branches in the deep part of the phylogeny (Supplementary Fig. S2). Instead, the datasets constructed by TADA cover a larger diversity of the *Planctomycetota* phylum, where both datasets include 23 of the classes (Fig. 1C). The phylogenies constructed from the taxonomic sampling (Supplementary Fig. S3) and phylogenomic sampling (Supplementary Fig. S4) have more and shorter branches close to the root compared with the phylogeny based on the random sampled dataset (Fig. 1D). In the TADA sampling, the only classes missing were those where no high-quality genome was available. Whereas the phylogenies constructed from the TADA-sampled datasets share the same overall topology, the topology of the phylogeny from the randomly sampled dataset differs. The most notable difference is the class UBA8742 which forms a clade with other classes in this phylogeny compared to phylogenies from the TADA-constructed datasets. Furthermore, the Colless indexes (Ci) (Colless and Wiley 1982) for the three phylogenies show that taxonomic sampling generates the most balanced phylogeny (Ci = 272), followed by phylogenomic sampling (Ci = 314), and random sampling (Ci = 338).

## 5 Conclusion

TADA can be used as a first step in evolutionary and comparative genomic studies to easily assemble robust and balanced genome datasets. Compared to phyloSkeleton, designed for a similar purpose, TADA is significantly easier to install, provides a more user-friendly interface, a higher granularity in how taxa should be sampled, and the possibility to work directly with GTDB’s phylogenies. It also conveniently prepares the following steps by preparing BLAST databases. Implementation in snakemake ensures reproducibility and the possibility to extend the workflow with additional downstream steps.

## Supplementary Material

btad742_Supplementary_Data

## Data Availability

Phylogenies and alignments underlying Fig. 1D are available from https://doi.org/10.17044/scilifelab.24106149.v1.
