# Supplementary material for: TADA: taxonomy-aware dataset aggregator
Source: Bioinformatics. 2023 Dec 7;39(12):btad742. doi: 10.1093/bioinformatics/btad742 (PMC10733731; doi:10.1093/bioinformatics/btad742)

Supplementary Information

Methods for usage example

Two datasets for the *Planctomycetota* phylogeny were constructed with TADA. The first was constructed using the taxonomic-based sampling approach. A sampling scheme was created based on the GTDB r214 (Parks et al. 2022) taxonomy and was defined to sample at most five species with a completeness estimate of $\geq 90\%$ and a contamination estimate of $\leq 5\%$ (high-quality genomes) from each class in the *Planctomycetota* phylum. As outgroup, we sampled ten species from the *Chlamydiota* phylum and ten species from the *Kiritimatiellae*, *Lentisphaeria*, and *Verrucomicrobiae* classes, respectively. This resulted in a dataset with 76 species from *Planctomycetota*. A second dataset was constructed using the phylogenomic-based sampling approach in TADA. The *Planctomycetota* phylum in the GTDB r214 reference phylogeny was pruned until 76 high-quality species remained. Finally, a third dataset was created by randomly sampling 76 high-quality *Planctomycetota* genomes. The proteomes for all sampled species were downloaded through TADA, and the proteomes for the outgroup species from the taxonomic-based dataset were added to the two other datasets.

A phylogeny was calculated for each dataset with the following method; HMMs for marker genes were downloaded from GTDB and hmmsearch from HMMER v3.3.2 (Eddy 2011) was used to search the proteomes. Based on this, the best hit for each species and marker gene was extracted. Extracted marker genes were aligned with MAFFT v7.471 using the L-INS-I option (Katoh and Standley 2013), and the alignments were concatenated using PhyKIT v.1.11.14 (Steenwyk et al. 2021). Sites with more than 90% gaps were removed using TrimAl v.1.4.1 (Capella-Gutiérrez, Silla-Martínez and Gabaldón 2009) and a phylogeny was calculated using IQ-Tree v2.2.0 (Minh et al. 2020) under the LG+F+G model with 1000 ultrafast bootstrap replicates (Hoang et al. 2018) and 1000 SH-aLRT replicates (Guindon et al. 2010).

Supplementary Figures

Supplementary Figure 1. Comparison of execution times for pruning the GTDB r214 archaeal phylogeny using Treemmer (orange) and TADA (blue).

Supplementary Figure 2. Maximum likelihood phylogeny of the *Planctomycetota* phylum from a randomly sampled dataset. Branch support values are indicated on the branches with SH-aLRT and ultrafast bootstrap support, respectively.

Supplementary Figure 3. Maximum likelihood phylogeny of the *Planctomycetota* phylum from a dataset constructed by the taxonomic sampling approach in TADA. Branch support values are indicated on the branches with SH-aLRT and ultrafast bootstrap support, respectively.

Supplementary Figure 4. Maximum likelihood phylogeny of the *Planctomycetota* phylum from a dataset constructed by the phylogenomic sampling approach in TADA. Branch support values are indicated on the branches with SH-aLRT and ultrafast bootstrap support, respectively.

References

Capella-Gutiérrez, Salvador, Silla-Martínez, José M., and Gabaldón, Toni, ‘trimAl: A Tool for Automated Alignment Trimming in Large-Scale Phylogenetic Analyses’, *Bioinformatics* , 25/15 (2009), 1972–73

Eddy, Sean R., ‘Accelerated Profile HMM Searches’, *PLoS Computational Biology*, 7/10 (2011), e1002195

Guindon, Stéphane, Dufayard, Jean-François, Lefort, Vincent, Anisimova, Maria, Hordijk, Wim, and Gascuel, Olivier, ‘New Algorithms and Methods to Estimate Maximum-Likelihood Phylogenies: Assessing the Performance of PhyML 3.0’, *Systematic Biology*, 59/3 (2010), 307–21

Hoang, Diep Thi, Chernomor, Olga, von Haeseler, Arndt, Minh, Bui Quang, and Vinh, Le Sy, ‘UFBoot2: Improving the Ultrafast Bootstrap Approximation’, *Molecular Biology and Evolution*, 35/2 (2018), 518–22

Katoh, K., and Standley, D. M., ‘MAFFT Multiple Sequence Alignment Software Version 7: Improvements in Performance and Usability’, *Molecular Biology and Evolution*, 30/4 (2013), 772–80

Minh, Bui Quang, Schmidt, Heiko A., Chernomor, Olga, Schrempf, Dominik, Woodhams, Michael D., von Haeseler, Arndt, et al., ‘IQ-TREE 2: New Models and Efficient Methods for Phylogenetic Inference in the Genomic Era’, *Molecular Biology and Evolution*, 37/5 (2020), 1530–34

Parks, Donovan H., Chuvochina, Maria, Rinke, Christian, Mussig, Aaron J., Chaumeil, Pierre-Alain, and Hugenholtz, Philip, ‘GTDB: An Ongoing Census of Bacterial and Archaeal Diversity through a Phylogenetically Consistent, Rank Normalized and Complete Genome-Based Taxonomy’, *Nucleic Acids Research*, 50/D1 (2022), D785–94

Steenwyk, Jacob L., Buida, Thomas J., Labella, Abigail L., Li, Yuanning, Shen, Xing-Xing, and Rokas, Antonis, ‘PhyKIT: A Broadly Applicable UNIX Shell Toolkit for Processing and Analyzing Phylogenomic Data’, *Bioinformatics (Oxford, England)*, 37/16 (2021), 2325–31

Supplementary Figure 1

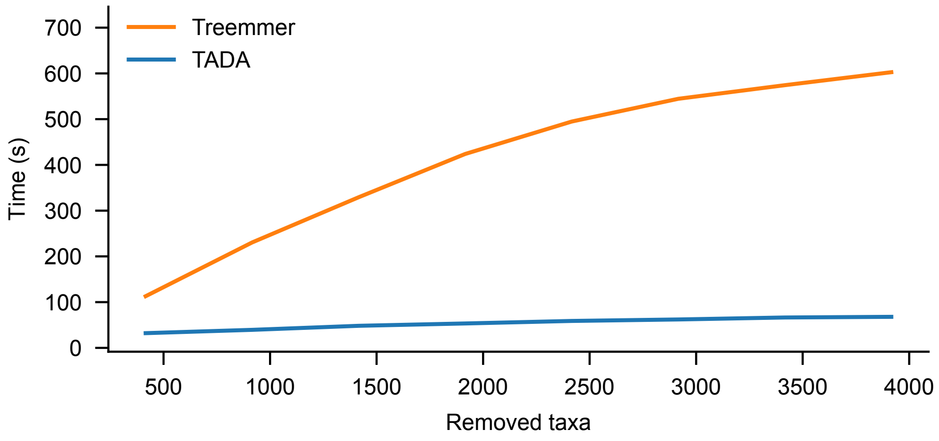

Supplementary Figure 2

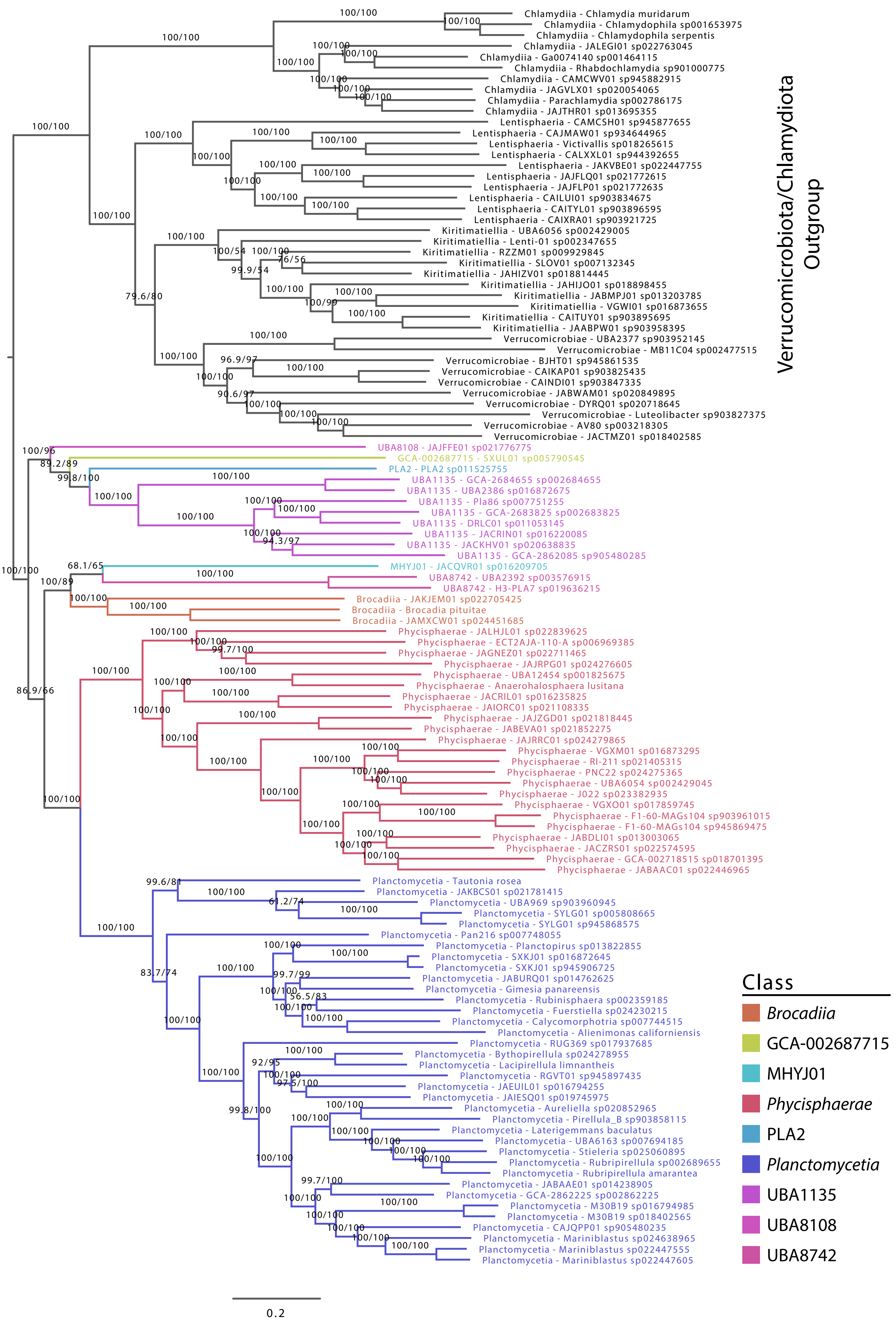

Supplementary Figure 3

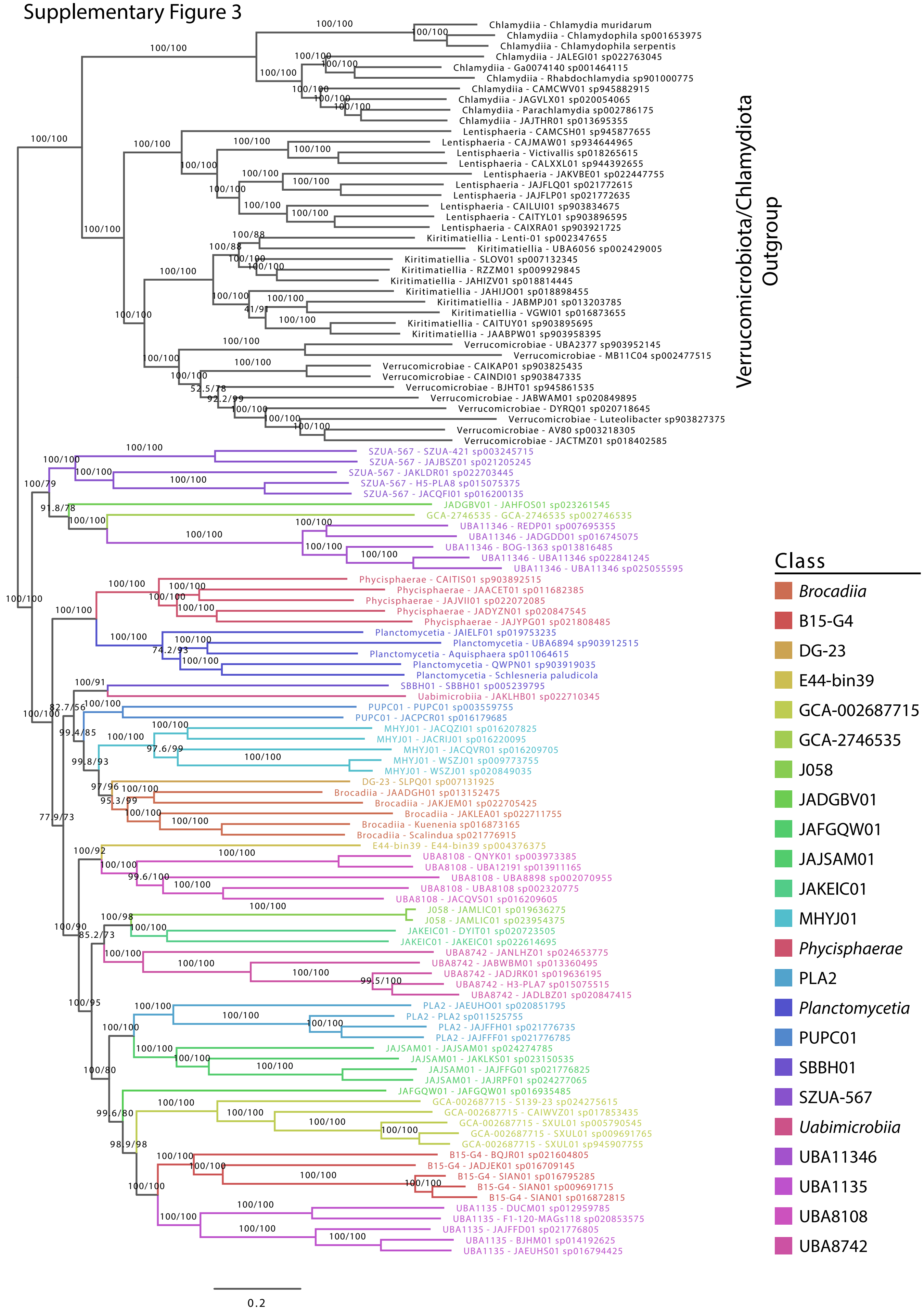

Supplementary Figure 4

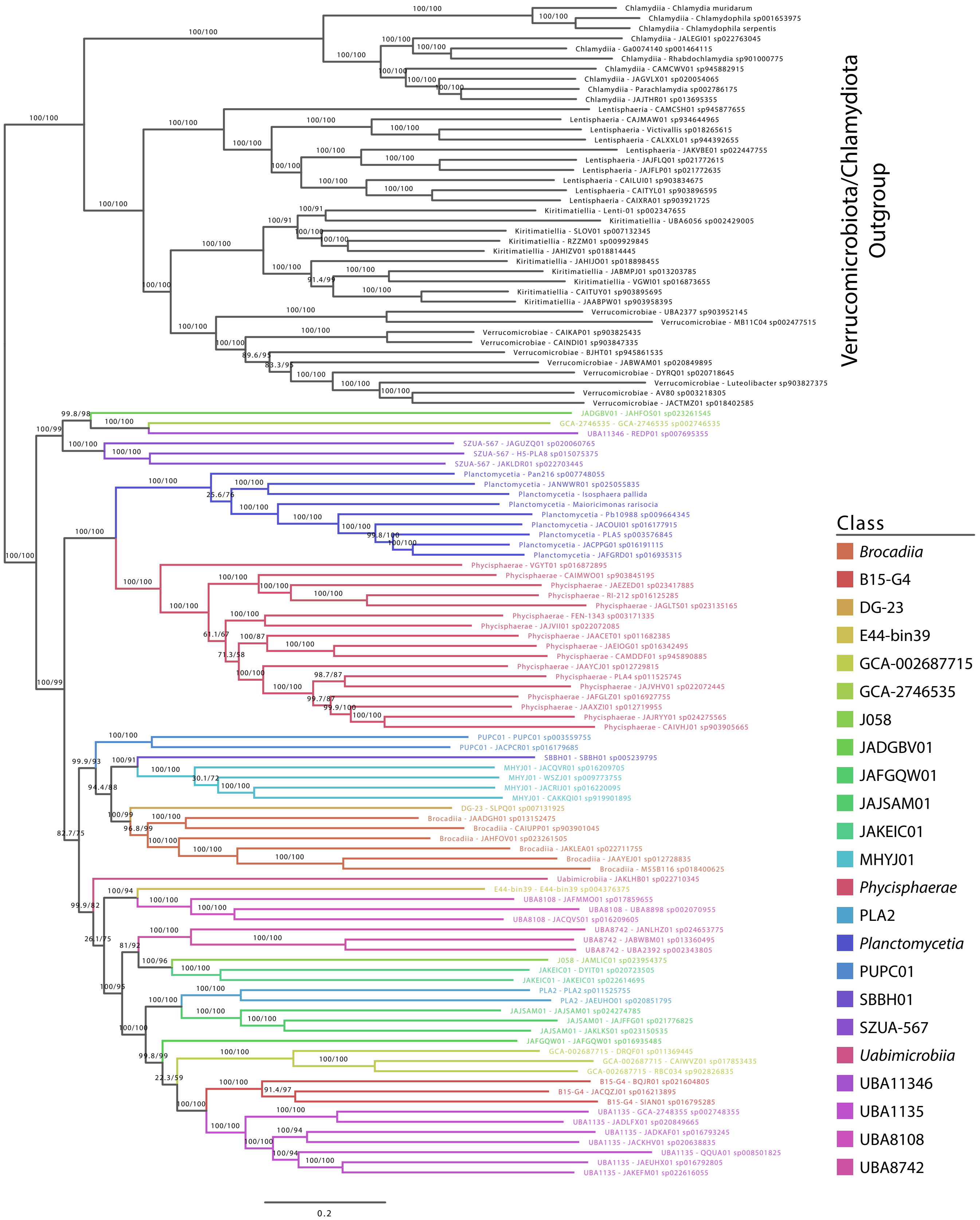

Supplement: btad742_Supplementary_Data [file btad742_supplementary_data.pdf]
